# Measuring the felt sense of dehumanization: A COSMIN systematic review of the psychometric properties of self‐ and meta‐dehumanization measures

**DOI:** 10.1111/bjop.70017

**Published:** 2025-08-08

**Authors:** Tom A. Jenkins, Hannah Pendlebury, Spencer L. Smith

**Affiliations:** ^1^ Department of Psychology University of Bath Bath UK; ^2^ Sirona Care & Health Bristol UK; ^3^ University Hospitals Bristol and Weston NHS Foundation Trust Bristol UK

**Keywords:** COSMIN, meta‐dehumanization, self‐dehumanization, self‐infrahumanization; psychometric, self‐objectification

## Abstract

There is increasing awareness of the clinical relevance of self‐ and meta‐dehumanization. With various measures available for use, evidence of robust reliability and validity is essential before implementation. This review aimed to evaluate the psychometric strength and methodological quality of self‐ and meta‐dehumanization measures and make recommendations for practice using Consensus‐based Standards for the selection of health Measurement Instruments (COSMIN) guidance. A systematic search of Web of Science, PubMed, PsycINFO and Scopus was conducted to identify studies reporting on the development or validation of a measure of self‐ or meta‐dehumanization. Of 5190 records, 26 studies containing 29 distinct outcome measures were identified (14 self‐dehumanization and 15 meta‐dehumanization). In general, there was a lack of involvement from people with lived experience in measure development, leading to very low quality of evidence for content validity. Strength and quality of other psychometric properties varied, with only some measures demonstrating sufficient high‐quality ratings. Based on COSMIN guidance, only one measure, the Experience of Dehumanization Measure (Golossenko et al., *Br. J. Soc. Psychol*., *62*, 2023, 1285), can be currently recommended for use. It is recommended that future research looks to: (1) improve efforts to validate existing measures and (2) develop gold standard measures in collaboration with people with lived experience.

## BACKGROUND

The felt sense of dehumanization is a growing area of research interest. Theoretically, dehumanization is the perception of another person as less or other than human, and has been extensively explored in psychological research (see Haslam, 2006; Haslam & Loughnan, 2014 for reviews). The victim's perspective of dehumanization is categorized as meta‐dehumanization and self‐dehumanization. Meta‐dehumanization refers to an individual's perception that they, or a group they belong to, are seen as less than human by others (Bastian & Haslam, 2010; Kteily et al., 2016). Self‐dehumanization refers to one's perception that they themselves are less than fully human (Bastian & Crimston, 2014). Although recently the ‘experience of dehumanization’ has been proposed as a distinct third type of dehumanization (Golossenko et al., 2023), it seems to be an elaboration of the meta‐dehumanization construct, focusing on the perception of how others ‘treat me’ rather than view me. We, therefore, propose that together, meta‐ and self‐dehumanization adequately conceptualize the ‘felt sense of dehumanization’.

Meta‐dehumanization and self‐dehumanization have largely been explored within social contexts to date, including university and workplace settings and within romantic relationships (Baldissarri et al., 2014; Bastian & Haslam, 2010, 2011; Caesens et al., 2017; Pizzirani et al., 2019). However, there is an emerging body of evidence recognizing the felt sense of dehumanization within clinical populations. Firstly, research has found that people diagnosed with severe alcohol use disorders (SAUD) often feel dehumanized by others (i.e. meta‐dehumanization; Fontesse et al., 2020), and are likely to experience self‐dehumanization when they are in agreement with the stigma directed towards them (Fontesse, Stinglhamber, et al., 2021). Self‐dehumanization is experienced by voice hearers; participants in a qualitative study described it as the end of a continuum constituting feelings of reduced autonomy, self‐worth, belonging, trust in oneself, sense of self and distressing sensory fragmentation (O'Brien‐Venus et al., 2023). The felt sense of dehumanization has not only been observed in these clinical populations, but has also been associated with a range of other clinical outcomes. For instance, meta‐dehumanization is associated with feelings of anger, sadness, shame and cognitive deconstructive states including impaired thought clarity and emotional numbing (Bastian & Haslam, 2011; Zhang et al., 2017). In a sample of people diagnosed with SAUD, meta‐dehumanization was moderately associated with anxiety, depression and reduced self‐efficacy around alcohol refusal, with self‐dehumanization mediating the links between these variables (Fontesse, Demoulin, et al., 2021). Self‐dehumanization was also moderately associated with anxiety, negative affect and physical symptoms of distress in a non‐clinical population (Sakalaki et al., 2017).

Given the clinical relevance of self‐ and meta‐dehumanization, outcome measures are required to further explore their causes, consequences, associations and possible interventions across different populations (Jenkins et al., 2023). Mokkink et al. (2010) emphasizes the need for valid and reliable health‐related patient‐reported outcome measures in order to obtain clinically accurate and relevant information in these settings. Health‐related outcome measures are therefore expected to meet particular standards with regard to their methodological and psychometric quality, and are evaluated with tools such as the Consensus‐based Standards for the selection of health Measurement Instruments (COSMIN) checklist (Mokkink, Prinsen, et al., 2018). COSMIN appraises psychometric strength and methodological quality of outcome measures, guiding evidence‐based recommendations for practice. This is achieved through evaluating various aspects of reliability and validity. If self‐ and meta‐dehumanization are to be considered clinical outcomes, measures must be held to the same rigour and quality standards as other health‐related outcome measures.

However, there are a number of criticisms of outcome measures currently used within health research and clinical practice. First, there has been a considerable increase in the number of outcome measures made available in recent years, making it difficult to identify the most suitable measure (Fleischmann & Vaughan, 2018). This is particularly true when multiple measures exist for the same health construct, resulting in a lack of clarity around which is best. Furthermore, many health‐related outcome measures exist which either report poor psychometric properties, or do not report them at all, which runs the risk of treatment effects being overestimated (Gagnier & Johnston, 2019). Several systematic reviews have also found variations in the methodological and psychometric quality of measures within mental health contexts when compared against quality assessment criteria (Breedvelt et al., 2020; El‐Den et al., 2018; Smith et al., 2021). Accordingly, a systematic review of the measures that exist for self‐ and meta‐dehumanization is required to identify whether similar methodological and psychometric issues are present in the measurement of these phenomena.

### Review aim

The aim of this review is to identify, summarize and quality assess the psychometric properties and methodological quality of all distinct self‐report measures of self‐ and meta‐dehumanization across all populations. To achieve this, all studies in which a measure of self‐or meta‐dehumanization is used will be identified, as well as each distinct measure used within these studies. The psychometric properties of these distinct measures will then be quality assessed against the COSMIN guidance (Mokkink, Prinsen, et al., 2018). Based upon this assessment and synthesis of findings, we will then make recommendations for best practice for any future measures of the felt sense of dehumanization.

## METHOD

This review is linked to a protocol registered on the Open Science Framework (https://osf.io/6er4t). The protocol stated that the psychometric properties of each measure would be explicitly reported. However, it was decided to instead follow the recommendation of Terwee et al. (2007) to present an overview of all quality assessment ratings (e.g. +, ?, −) rather than raw coefficients (e.g. *⍺* = .72), as the objective was to provide an overall appraisal of measurement quality rather than a meta‐analysis of numerical estimates (Table 2). This change was made after full‐text screening and before any data extraction or quality assessment had begun.

The review was reported following Preferred Reporting Items for Systematic reviews and Meta‐Analyses (PRISMA) guidelines (Page et al., 2021). A table displaying guidelines and where each item can be found within the manuscript is presented in the Supporting Information.

### Search strategy

Web of Science, PubMed, PsycINFO and Scopus were systematically searched for all records, with no lower limit set for starting year. Searches were first run on 23 February 2024, then re‐run on 27 September 2024 and 23 January 2025. Grey literature was not included given the nature of the review, in line with other systematic reviews of outcome measures (eg. Smith et al., 2021). A search strategy was developed and adapted for each database (see Supporting Information). The search terms included: dehuman* OR infrahuman* OR ‘internalizing objectification’, with the Boolean operator ‘OR’ used to search for words within one concept.

### Eligibility

The inclusion criteria were as follows: studies which develop or validate a self‐report measure of self‐ or meta‐dehumanization; full text available in a peer‐reviewed journal; accessible in the English language. The exclusion criteria were as follows: review articles; study protocols; grey literature; studies that did not use a self‐report measure; non‐human populations; studies referring to sexual self‐objectification. Once studies were identified as eligible, each distinct measure was extracted for quality assessment.

### Procedure

Following the database searches, identified references were exported to Covidence where duplicates were removed. The first step of the selection process involved screening the titles and abstracts of the identified studies for relevance. This was completed by two reviewers, who displayed substantial agreement. The full texts of the resulting articles were then reviewed for eligibility by two reviewers, again with high agreement. For the few discrepancies that emerged, a consensus was reached following discussions between reviewers regarding their decisions. Additional hand searches of the reference lists of eligible articles were not undertaken.

From these eligible studies, all distinct measures of the felt sense of dehumanization were extracted by identifying the study in which it was initially conceived. In keeping with COSMIN guidance, all instances of subsequent validation would be considered when evaluating the psychometric properties of each distinct measure.

### Data extraction

Information extracted from the original measure development papers included: measure name, administration method, number of items, theoretical conceptualization, scale type and target population.

### Quality assessment

The strength of the psychometric properties and methodological quality for each measure was extracted and assessed based on COSMIN guidance (Mokkink, Prinsen, et al., 2018). These properties included:

- Content validity – the extent to which the items represent the concepts of interest, encompassing item relevance, comprehensiveness and comprehensibility. COSMIN considers any content validity studies conducted with patients and/or professionals, and asks the authors of the review to also rate the content validity of each measure.
- Internal consistency – the degree to which items within a measure are similar, thereby measuring the same concept.
- Structural validity – whether similar item scores cluster together to reflect the dimensionality of the measure.
- Criterion validity – the degree to which scores relate to a ‘gold standard’ measure.
- Hypothesis testing for construct validity – the extent to which scores relate to other measures in line with theory‐driven hypotheses linked to relevant concepts. It is acknowledged that not all authors conducted formal tests of construct validity (e.g. convergent/divergent correlations or known group differences). For this review, it was deemed that construct validity could be assessed through evaluation of any form of hypothesis testing within a study where a measure is first developed.
- Reliability (here meaning test retest) – referring to the consistency of results when the measure is repeated over a short period of time.
- Measurement invariance – whether scoring equivalence is demonstrated across group factors in which no important differences are expected to exist (e.g. age, gender, cross‐culturally).
- Measurement error – changes in outcome measure score not owing to a change in outcome.
- Responsiveness – the ability of a measure to detect change over time.

Methodological quality of studies in which a measure was developed or validated was first assessed using the Risk of Bias checklist (Mokkink, De Vet, et al., 2018). Quality was rated as either ‘very good’, ‘adequate’, ‘doubtful’, ‘inadequate’ or ‘not applicable’ based upon pre‐defined criteria set by Mokkink, Prinsen, et al. (2018). Two reviewers quality assessed all measures, with 88.7% agreement. Conflicts were discussed between the reviewers who mutually agreed upon a rating.

The quality of the psychometric properties was subsequently rated with the COSMIN criteria for good measurement properties (Mokkink et al., 2024). Each property is given a ‘sufficient’, ‘indeterminate’ or ‘insufficient’ rating, incorporating information about the strength of the property and some elements of methodology. All data were extracted and rated against the COSMIN Criteria by two reviewers, with a 92.5% agreement rate indicated between reviewers. In the case of content validity, COSMIN asks reviewers to consider patient, professional and reviewer ratings when summarizing a score. Two reviewers rated the content validity for all studies with a 90.8% agreement. Conflicts were discussed between the reviewers who agreed upon a rating for each instance.

Finally, the Grading of Recommendations, Assessment, Development and Evaluation (GRADE) approach was used to determine overall evidence quality. GRADE assumes ‘High’‐quality evidence, and studies can be downgraded to ‘Moderate’, ‘Low’ or ‘Very Low’ based upon Risk of Bias (poor study quality), inconsistency (multiple different findings), imprecision (low sample sizes) and indirectness (evidence coming from differing samples). Two reviewers calculated all GRADE ratings with 93.1% agreement. Conflicts were discussed between reviewers who agreed upon a rating for each instance.

## RESULTS

**FIGURE 1:**
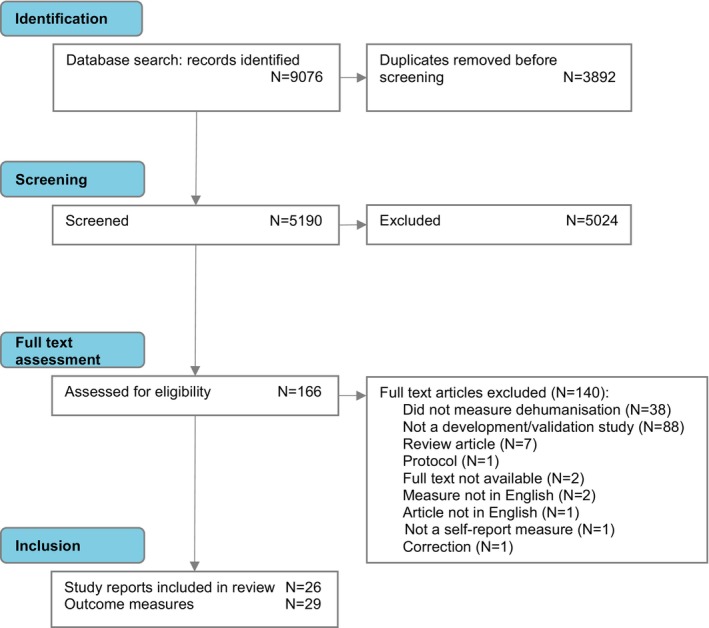
PRISMA‐COSMIN flow diagram of search and selection process.

Figure 1 displays a PRISMA‐COSMIN for Outcome Measure Instruments (OMIs) (Elsman et al., 2024) flow diagram summarizing the search and selection process. A total of 9076 records were identified from database searches. Covidence removed 3892 duplicates, leaving 5190 articles which underwent title and abstract screening. In total, 166 full‐text articles were reviewed, with 140 excluded overall for the following reasons: article did not measure the felt sense of dehumanization; article did not develop or validate a measure of the felt sense of dehumanization; review article; study protocol; full text not available (despite contacting authors); measures not in English language; not a self‐report measure; correction to an article; and full text not in the English language. Six further studies measuring the felt sense of dehumanization which had not appeared during the initial database search were identified during full‐text screening, appearing within methodology sections in which the studies were described as contributing to the development of other measures.

Overall, 26 studies developing or validating a measure of the felt sense of dehumanization were eligible for inclusion. Included within these were 29 original measures of the felt sense of dehumanization. Although not all measures were explicitly described in terms of ‘self‐dehumanization’ and ‘meta‐dehumanization’, 14 measures appeared to comprise items that assessed the concept of self‐dehumanization (i.e. an individual's perception that they are less or other than fully human) and 15 measures comprised items that assessed meta‐dehumanization (i.e. an individual's perception that they, or a group they belong to, are seen as less or other than human by others). The majority of studies described the instances in which an original measure was developed; only one study conducted subsequent validation of a measure (Roupa et al., 2024). Table 1 displays a summary of all measures. Table 2 displays a summary of psychometric strength and quality assessment of all measures. A table of all included studies can be found in Supporting Information.

**TABLE 1:** Characteristics of self‐ and meta‐dehumanization measures.

Measure name and original author	*N* items	Theoretical conceptualization	Likert scale type	Target population
Self‐dehumanization measures				
Self‐dehumanization measure (Bastian & Haslam, 2010)	12	Human nature & human uniqueness^a^	7‐point	General population
Measure of self‐humanity (Bastian et al., 2013)	8	Human nature & human uniqueness^a^	7‐point	General population
Self‐perception of being instrument‐like vs. human‐like in the workplace measure (Baldissarri et al., 2017)	10	Instrument‐like & human‐like^b^	7‐point	Workers
Self‐mental state attribution task in the workplace (Baldissarri et al., 2014)	20	Perceptions, wishes, thoughts, intentions^c^	7‐point	Workers
Self‐dehumanization scale in patients with severe alcohol use disorder (Fontesse, Demoulin, et al., 2021)	13	Human nature & human uniqueness^a^	7‐point	Patients with severe alcohol use disorder
Self‐dehumanization measure adapted from mind attribution scale (Kouchaki et al., 2018)^i^	10	Emotion, intention, cognition^d^	7‐point	General population
Mechanistic self‐dehumanization scale (Sakalaki et al., 2017)	14	Human nature^a^	9‐point	General population and healthcare workers
Self‐objectification scale (Talmon & Ginzburg, 2016)	17	Invisibility & lack of autonomy	5‐point	General population
Self‐infrahumanization scale in African American women (McCleary‐Gaddy & James, 2022)	16	Primary and secondary emotions^e^	5‐point	African American women
Low human nature traits scale (Sakalaki et al., 2016)	5	Human nature^a^	9‐point	General population
Self‐Dehumanization Scale (SDS) (Robison et al., 2025)	8	Animalistic & mechanistic^h^	7‐point	General (non‐clinical) population, people with self‐disclosed mental health diagnosis, people with a minoritized identity
Patient gown dehumanization questionnaire (Punchihewa & Broadbent, 2024)	11	None mentioned	5‐point	Hospital patients
Self‐infrahumanization from sexism (Cervone et al., 2025)	10	Primary and secondary emotions^f^	7‐point	Women
Self‐objectification from sexism (Cervone et al., 2025)	9	Instrument‐like & human‐like^b^	7‐point	Women
Meta‐dehumanization measures				
Organizational dehumanization measure (Caesens et al., 2017)^j^	11	Organizational dehumanization	7‐point	Workers
Organizational dehumanization measure short (Lagios et al., 2024)	5	Organizational dehumanization	7‐point	Workers
Meta‐dehumanization measure (Kteily et al., 2016)	5/6	None	6/7‐point	Groups involved in intergroup conflict, for example, Israeli and Palestinian populations
Meta‐dehumanization scale in patients with severe alcohol use disorder (Fontesse et al., 2020)	13	Human nature & human uniqueness^a^	7‐point	Patients with severe alcohol use disorder
Meta‐dehumanization measure (Bastian & Haslam, 2011)	10	Human nature & human uniqueness^a^	6‐point	General population
Meta‐dehumanization in women measure (Demoulin et al., 2021)	12	Human nature & human uniqueness^a^	7‐point	Women
Perception of being objectified by supervisors (PBOS; Baldissarri et al., 2014)	9	Instrumentality, denial of autonomy, inertness, fungibility, violability, ownership, denial of subjectivity^g^	7‐point	Workers
Perception of being objectified in the workplace scale (Auzoult & Personnaz, 2016)	26	Instrumentalization, reduction to appearance, reduction to body, reduction to silence, denial of autonomy, denial of subjectivity, passivity, interchangeability, violability, possession	7‐point	Workers
Perception of objectification in the workplace short scale (POWS; Crone & Brunel, 2021)	10	Instrumental value & powerfulness	7‐point	Workers
Meta‐dehumanization in people with severe alcohol use disorder measure (Demoulin et al., 2021)	19	Human nature & human uniqueness^a^	7‐point	Patients with severe alcohol use disorder
Experience of dehumanization measure (Golossenko et al., 2023)	10	Experience of dehumanization	5‐point	General population
Dehumanization within romantic relationships scale (Pizzirani et al., 2019)	12	Immature, unrefined, exploitable and emotionless^h^	7‐point	People in a romantic relationship
Measure of perceived dehumanization from officers (Robison et al., 2024)	3	Animalistic, stupid, loss of identity	4‐point	People incarcerated in prisons and jail
Organizational animalistic dehumanization scale (Cheung, 2024)	10	Animalistic dehumanization	5‐point	Full time workers
Meta‐dehumanization from sexism (Cervone et al., 2025)	6	None: ‘ad hoc’	7‐point	Women

^a^ Dual model of dehumanization, Haslam (2006).

^b^ Adapted from measure of instrumentality, Andrighetto et al. (2017).

^c^ A folk model of the mind, D'Andrade (1987), adapted by Haslam et al. (2008).

^d^ Mind attribution, Kouchaki et al. (2018).

^e^ Infrahumanization, Leyens et al. (2000, 2001).

^f^ Objectification, Gruenfeld et al. (2008).

^g^ Dual model of dehumanization, Haslam (2006) and Pizzirani and Karantzas (2019).

^h^ Animalistic and mechanistic items drawn from variety of empirical theory, primarily Haslam (2006) and Bandura et al. (1975).

^i^ Chinese translation available (Jiang et al., 2021).

^j^ Spanish and Arabic translations available (Abou Zeid et al., 2024; Ariño‐Mateo et al., 2022).

**TABLE 2:** Psychometric property strength, GRADE assessment of evidence quality and Risk of Bias for each measure of self‐ and meta‐dehumanization.

Measure and original author	Psychometric strength						GRADE evidence quality (risk of bias)					
	Content validity	Internal consistency	Structural validity	Construct validity	Reliability	Measurement invariance	Content validity	Internal consistency	Structural validity	Construct validity	Reliability	Measurement invariance
Self‐dehumanization measures												
Self‐dehumanization measure (Bastian & Haslam, 2010)	+	?		+			Very low (1)	Moderate (4)		Moderate (4)		
Measure of self‐humanity (Bastian et al., 2013)	±	+	−	+			Very low (1)	Moderate (4)	Low (2)	Moderate (4)		
Self‐perception of being instrument‐like vs. human‐like in the workplace measure (Baldissarri et al., 2017)	±	?		+			Very low (1)	High (4)		High (4)		
Self‐mental state attribution task in the workplace (Baldissarri et al., 2014)	−	?		+			Very low (1)	High (4)		High (4)		
Self‐dehumanization scale in patients with severe alcohol use disorder (Fontesse, Demoulin, et al., 2021)	±	?		+			Very low (1)	High (4)		High (4)		
Self‐dehumanization measure adapted from mind attribution scale (Kouchaki et al., 2018)	±	?		+			Very low (1)	High (4)		High (4)		
Mechanistic self‐dehumanization scale (Sakalaki et al., 2017)^a^	±	+	+	+	+		Doubtful (1)	High (4)	Moderate (3)	High (4)	Low (2)	
Self‐objectification scale (Talmon & Ginzburg, 2016)	±	+	−	+			Very low (1)	High (4)	High (4)	High (4)		
Self‐infrahumanization scale in African American women (McCleary‐Gaddy & James, 2022)	−	?		−			Very low (1)	High (4)		High (4)		
Low human nature traits scale (Sakalaki et al., 2016)	−	?		+			Very low (1)	High (4)		High (4)		
Self‐Dehumanization Scale (SDS) (Robison et al., 2025)	±	+	+	+			Very low (1)	High (4)	High (4)	High (4)		
Patient gown dehumanization questionnaire (Punchihewa & Broadbent, 2024)	±	?		+			Very low (1)	Moderate (4)		Moderate (4)		
Self‐infrahumanization from sexism (Cervone et al., 2025)	±	?		+			Very low (1)	High (4)		High (4)		
Self‐objectification from sexism (Cervone et al., 2025)	±	?		+			Very low (1)	High (4)		High (4)		
Meta‐dehumanization measures												
Organizational dehumanization measure (Caesens et al., 2017)	+	+	+	+			Very low (1)	High (4)	High (4)	High (4)		
5‐item organizational dehumanization measure short (Lagios et al., 2024)	+	+	+	+			Very low (1)	High (4)	High (4)	High (4)		
Meta‐dehumanization measure (Kteily et al., 2016)	±	?		+			Very low (1)	High (4)		High (4)		
Meta‐dehumanization scale in patients with severe alcohol use disorder (Fontesse et al., 2020)	±	?		+			Very low (1)	High (4)		High (4)		
Meta‐dehumanization measure (Bastian & Haslam, 2011)	+	+	+	+			Very low (1)	Moderate (4)	Low (2)	Moderate (4)		
Meta‐dehumanization in women measure (Demoulin et al., 2021)	±	+	+	+			Very low (1)	High (4)	Moderate (3)	High (4)		
Perception of being objectified by supervisors (PBOS) (Baldissarri et al., 2014)	−	?		+			Very low (1)	High (4)		High (4)		
Perception of being objectified in the workplace scale (Auzoult & Personnaz, 2016)	±	+	−	+			Very low (1)	Low (2)	Moderate (3)	High (4)		
Perception of objectification in the workplace short scale (POWS; Crone & Brunel, 2021)	+	+	+	+			Very low (1)	High (4)	High (4)	High (4)		
Meta‐dehumanization in people with severe alcohol use disorder measure (Demoulin et al., 2021)	±	+	+	+			Very low (1)	High (4)	Moderate (3)	High (4)		
Experience of dehumanization measure (Golossenko et al., 2023)	+	+	+	+		+	High (4)	High (4)	High (4)	High (4)		High (4)
Dehumanization within romantic relationships scale (Pizzirani et al., 2019)	+	+	+	+			Very low (1)	High (4)	High (4)	High (4)		
Measure of perceived dehumanization from officers (Robison et al., 2024)	+	+	+	+			Very low (1)	High (4)	High (4)	High (4)		
Organizational animalistic dehumanization scale (Cheung, 2024)	−	+	+	+	+		Very low (1)	High (4)	High (4)	High (4)	Low (2)	
Meta‐dehumanization from sexism (Cervone et al., 2025)	±	?		+			Very low (1)	High (4)		High (4)		

*Note*: For psychometric strength: + = sufficient rating; ? = indeterminate rating; − = insufficient rating; ± = inconsistent rating. Risk of Bias ratings are indicated in brackets after GRADE evidence quality ratings: 1 = inadequate, 2 = doubtful, 3 = adequate, 4 = very good.

^a^ Pooled ratings are presented.

### Self‐dehumanization measures

Of the 14 self‐dehumanization measures identified, the number of items ranged from five to 20. Scale types ranged from five‐point to nine‐point. With regard to the theoretical concepts drawn upon to develop the items, five measures were based upon human nature and human uniqueness taken from the Dual Model of Dehumanization (Haslam, 2006), with two of these measures only using the element of human nature. Two measures were based on the concepts of being instrument‐like and human‐like (Andrighetto et al., 2017). One measure drew upon the concepts of perceptions, wishes, thoughts and intentions (Haslam et al., 2008). One measure was based upon the concepts of emotion, intention and cognition, taken from the theory of mind attribution (Kouchaki et al., 2018). One measure created its own concepts of invisibility and lack of autonomy (Talmon & Ginzburg, 2016). One measure (Robison et al., 2025) was informed by animalistic and mechanistic concepts drawn from a variety of theories, primarily Haslam's (2006) notions and Bandura et al.'s (1975) animalistic descriptors. Two measures were conceptualized by primary and secondary emotions taken from infrahumanization theory (Leyens et al., 2000, 2001). Finally, one measure (Punchihewa & Broadbent, 2024) did not mention any theoretical underpinnings.

### Meta‐dehumanization measures

Of the 15 meta‐dehumanization measures identified, the number of items ranged from three to 26. Scale types ranged from four‐point to seven‐point. The theoretical conceptualization of four measures drew upon the Dual Model of Dehumanization (Haslam, 2006). One measure created its own theoretical concept of organizational dehumanization (Caesens et al., 2017), which was subsequently validated as a short form (Lagios et al., 2024). One measure did not draw upon any theoretical background (Kteily et al., 2016). One measure was conceptualized by instrumentality, denial of autonomy, inertness, fungibility, violability, ownership and denial of subjectivity, taken from objectification theory (Gruenfeld et al., 2008). One measure extended this using the concepts of instrumentalization, reduction to appearance, reduction to body, reduction to silence, denial of autonomy, denial of subjectivity, passivity, interchangeability, violability and possession (Auzoult & Personnaz, 2016). One measure used its own concepts of instrumental value and powerfulness (Crone & Brunel, 2021). One measure created its own theoretical construct, namely the experience of dehumanization (Golossenko et al., 2023). One measure used the concepts immature, unrefined, exploitable and emotionless, drawing upon the Dual Model of Dehumanization (Haslam, 2006) and research by Pizzirani and Karantzas (2019). One measure created theoretically informed concepts of animalistic, stupid and loss of identity (Robison et al., 2024). One measure (Cheung, 2024) drew upon animalistic concepts by Haslam (2006) and Bell and Khoury (2011). One measure (Cervone et al., 2025) did not mention theoretical underpinnings.

### Risk of bias: Quality assessment of methodology

The Experience of Dehumanization Measure (Golossenko et al., 2023) was the only measure to receive a ‘Very good’ rating for content validity. This is due to a rigorous development process involving a qualitative survey and semi‐structured interviews asking people about their experiences of dehumanization to generate items, followed by a consultation with professionals about the relevance, comprehensibility and comprehensiveness of the draft items. The Mechanistic Self‐Dehumanization Scale (Sakalaki et al., 2017) received a ‘Doubtful’ rating for content validity. In their initial development study, there was no input from professionals or the target population. In a subsequent validation study by Roupa et al. (2024), three dehumanization experts evaluated item relevance and 10 members of the target population (doctors and nurses) were asked to comment on item relevance, comprehension and comprehensibility. However, with only three professionals involved, minimal information provided about how these studies were conducted and no information provided about the results of the target population study, a ‘Doubtful’ rating was awarded. All other measures were rated ‘Inadequate’ for content validity. Talmon and Ginzburg (2016) consulted two experts for feedback on the relevance, comprehensiveness and comprehensibility of the Self‐Objectification Scale, along with three students on its comprehensibility. Sakalaki et al. (2016) conducted a pilot study in which 31 students were asked to rate 15 traits as low or high human nature, in order to inform the development of their Low Human Nature Scale. Caesens et al. (2017) consulted a professional for comprehensiveness, comprehensibility and relevance. Pizzirani et al. (2019) had two independent experts consider the face validity of a long list of items, assessing their relevance and reducing the number of items. Cheung (2024) generated 20 candidate items from the literature, then consulted six subject matter experts (academic staff and PhD students) on item relevance, seeking above 75% agreement for item retention. As the target population was not involved in determining the comprehensibility, comprehensiveness or relevance of items in any of these scales, all received an ‘Inadequate’ rating for content validity.

All but one measure was rated as' Very good' for internal consistency. The Perception of Being Objectified in the Workplace Scale (Auzoult & Personnaz, 2016) received a ‘Doubtful’ rating, as the dimensionality of the scale was not clear. Study quality for structural validity was mixed, with measures receiving ratings of ‘Very good’, ‘Adequate’ and ‘Doubtful’. Those receiving Doubtful ratings did so for an insufficient sample size and a lack of detail about rotation methods and suitability of the data for factor analysis. Those with ‘Adequate’ ratings did so for only conducting exploratory factor analysis and not confirmatory. All measures received a ‘Very good’ rating for construct validity. Test–retest reliability was assessed in two measures. The Mechanistic Self‐Dehumanization Scale (Sakalaki et al., 2017) and the Organizational Animalistic Dehumanization Scale (Cheung, 2024) received ‘Doubtful’ ratings, as correlations between timepoints were conducted without evidence that no systematic change has occurred. Measurement invariance was assessed in one measure (Golossenko et al., 2023) which received a ‘Very good’ rating. Risk of Bias ratings can be found in Table 2.

### Strength of psychometric properties

#### Content validity

All but one content validity rating was reviewers' ratings only, as no study apart from one reported data from content validity studies with professionals or target population. The exception to this is the Mechanistic Self‐Dehumanization Scale (MSDS) (Sakalaki et al., 2017), for which a professional's content validity was performed by Roupa et al. (2024). Content validity strength rating for the MSDS summarizes the professional study with reviewers' ratings.

##### Self‐dehumanization measures

One self‐dehumanization measure received a sufficient rating for content validity (Bastian & Haslam, 2010) as it was perceived to contain items which are comprehensible, comprehensive and relevant. Ten self‐dehumanization measures received inconsistent ratings for content validity. Three self‐dehumanization measures (Baldissarri et al., 2014; McCleary‐Gaddy & James, 2022; Sakalaki et al., 2016) received insufficient ratings for content validity, due to a perceived lack of comprehensiveness, comprehensibility and relevance.

##### Meta‐dehumanization measures

Seven meta‐dehumanization measures received sufficient ratings for content validity as they were perceived to contain items which are comprehensible, comprehensive and relevant (Bastian & Haslam, 2011; Caesens et al., 2017; Crone & Brunel, 2021; Golossenko et al., 2023; Lagios et al., 2024; Pizzirani et al., 2019; Robison et al., 2024). Six meta‐dehumanization measures received inconsistent ratings. Two meta‐dehumanization measures (Baldissarri et al., 2014; Cheung, 2024) received insufficient ratings for content validity due to a perceived lack of comprehensiveness, comprehensibility and relevance.

#### Internal consistency

##### Self‐dehumanization measures

Four measures of self‐dehumanization received sufficient ratings for internal consistency, due to having some evidence of factor structure and Cronbach's alpha > .70 (Bastian et al., 2013; Robison et al., 2025; Sakalaki et al., 2017; Talmon & Ginzburg, 2016). The remaining 10 measures received an indeterminate rating due to reporting Cronbach's alpha, but with a lack of information about scale dimensionality.

##### Meta‐dehumanization measures

Eleven measures of meta‐dehumanization received sufficient ratings for internal consistency, due to having some evidence of factor structure and Cronbach's alpha > .70. The remaining four measures received an indeterminate rating due to reporting Cronbach's alpha > .70, but with a lack of information about scale dimensionality (Baldissarri et al., 2014; Cervone et al., 2025; Fontesse et al., 2020; Kteily et al., 2016).

#### Hypothesis testing for construct validity

##### Self‐dehumanization measures

Thirteen measures received sufficient ratings for construct validity by testing convergent validity, finding more than 75% of results in line with their main hypotheses. Seven of these measures demonstrated construct validity throughout convergent validity tests. Six measures (Bastian et al., 2013; Bastian & Haslam, 2010; Cervone et al., 2025; Kouchaki et al., 2018; Punchihewa & Broadbent, 2024) demonstrated construct validity through subgroup comparison. One measure (McCleary‐Gaddy & James, 2022) received an insufficient rating, due to <75% of its results being in line with its hypotheses.

##### Meta‐dehumanization measures

All meta‐dehumanization measures received a sufficient rating for construct validity by testing convergent validity; more than 75% of their results were in line with their main hypotheses. Thirteen of these measures demonstrated construct validity throughout convergent validity tests. Two (Bastian & Haslam, 2011; Cervone et al., 2025) demonstrated construct validity through subgroup comparison.

#### Reliability (test–retest)

##### Self‐dehumanization measures

The Mechanistic Self‐Dehumanization Scale (Sakalaki et al., 2017) received a sufficient rating for reliability, as a test–retest correlation above .70 was found. Test–retest reliability was not conducted on the remaining 13 measures.

##### Meta‐dehumanization measures

The Animalistic Organization Dehumanization Scale (Cheung, 2024) received a sufficient rating for reliability, as a test–retest correlation above .70 was found. Test–retest reliability was not conducted on the remaining 14 measures.

#### Structural validity

##### Self‐dehumanization measures

Four self‐dehumanization measures underwent structural validity testing. Two (Robison et al., 2025; Sakalaki et al., 2017) received sufficient ratings for demonstrating evidence of good factor structure. Two (Bastian et al., 2013; Talmon & Ginzburg, 2016) received an insufficient rating due to their factor solutions explaining <50% of the variance. Eleven self‐dehumanization measures were not subject to structural validity tests.

##### Meta‐dehumanization measures

Ten meta‐dehumanization measures received a sufficient rating for structural validity. One measure (Auzoult & Personnaz, 2016) reports both a five‐factor solution with a substantial number of item cross‐loadings and a one‐factor solution explaining <50% of the variance; both iterations qualify for an insufficient rating. Four meta‐dehumanization measures did not conduct structural validity tests.

#### Measurement invariance

##### Self‐dehumanization measures

Measurement invariance was not assessed in any self‐dehumanization measure.

##### Meta‐dehumanization measures

Measurement invariance was sufficient in the only meta‐dehumanization measure which tested for it (Golossenko et al., 2023). Measurement invariance was not assessed in any other meta‐dehumanization measure.

### Other measurement properties

Measurement error and responsiveness were not assessed in any of the measures. Criterion validity was reported in some papers, but the comparison measures used were not deemed by the authors of this review to reflect the gold standard. These comparisons have instead been interpreted as evidence of construct validity.

### GRADE evidence

Table 2 presents ratings of overall evidence quality, with Risk of Bias ratings indicated alongside. These ratings were mostly influenced by Risk of Bias, and in four instances (Bastian et al., 2013; Bastian & Haslam, 2010, 2011; Punchihewa & Broadbent, 2024), sample size (imprecision). High Risk of Bias greatly influenced content validity, with all but two measures receiving a ‘very low’ rating. Construct validity, measurement invariance and internal consistency assessments were generally of ‘high’ quality evidence. Structural validity ratings were mixed. The two reliability assessments were ‘very low’ quality.

## DISCUSSION

This systematic review identified 26 studies in which a new measure of self‐ or meta‐dehumanization was used. Within these studies, 29 distinct self‐report questionnaires developed to measure aspects of the felt sense of dehumanization were identified: 14 measuring self‐dehumanization and 15 measuring meta‐dehumanization. There was one study (Roupa et al., 2024) in which a measure underwent further validation after its initial development. Quality assessment of the psychometric properties and methodological quality of these measures revealed their strengths and limitations, providing important information for researchers and clinicians regarding the ways in which the felt sense of dehumanization is currently measured.

None of the 24 measures explicitly measured both meta‐ and self‐dehumanization, meaning there is no current measure of the overall felt sense of dehumanization. This is surprising, as it has long been recognized how perceptions of negative other–self attitudes are commonly associated with negative self–self perceptions (Chadwick et al., 1999; Marquet et al., 2019; Neff & Vonk, 2009). It has however been acknowledged that meta‐ and self‐dehumanization are associated in the context of SAUD (Fontesse, Demoulin, et al., 2021), and a greater understanding of this relationship should be explored in future research. Not only could the concepts of meta‐ and self‐dehumanization apply to clinical populations, but also to any minoritized population in which dehumanization is known to occur, such as migrants and refugees (Hartley & Fleay, 2017; McLoughlin & Over, 2019), or where it is likely to continue to occur, such as the survivors of childhood sexual abuse, whereby research has suggested that societal stigmatization can often be internalized, resulting in self‐blame and shame (Kennedy & Prock, 2018). It would, therefore, be beneficial to research the wider application of the concepts of meta‐ and self‐dehumanization.

There is growing interest and understanding regarding the clinical relevance and implications of the felt sense of dehumanization (Bastian & Haslam, 2011; Fontesse, Demoulin, et al., 2021; O'Brien‐Venus et al., 2023; Zhang et al., 2017). Three measures are available for use by people with alcohol use disorder (Demoulin et al., 2021; Fontesse, Demoulin, et al., 2021; Fontesse et al., 2020). One measure (Robison et al., 2025) is validated in samples of students, people self‐reporting a mental health diagnosis and people with at least one minoritized identity. Alongside these, it is important that bespoke measures for specific client groups are developed. Terwee et al. (2007) argue that it is not appropriate to use a measure that has not been psychometrically validated within the representative population in question, as there may be specific factors within that population that compromise the validity or reliability of the measure. For instance, the subjective experience of self‐dehumanization for voice hearers documented by O'Brien‐Venus et al. (2023) may not be fully represented by a general self‐dehumanization measure. The development of any new measures should follow guidelines laid out by COSMIN (Mokkink et al., 2024) and ensure that people with lived experience are central to their development.

Based on COSMIN criteria, there is not a single self‐dehumanization measure that can be recommended for use. This is because no measure of self‐dehumanization has sufficiently high‐quality ratings for all measurement properties. Meta‐dehumanization measures were generally of higher quality. Based on the COSMIN criteria, only one meta‐dehumanization measure can be recommended for use: the Experience of Dehumanization measure (Golossenko et al., 2023), as it was the only measure with high‐quality evidence for its content, construct, structural validity, measurement invariance and internal consistency. However, as the authors identify, this measure specifically targets instances of dehumanizing treatment, rather than perception, which researchers choosing to use this measure should hold in mind. The lack of demonstrated psychometric validity of many of these measures calls into question the validity of existing self‐ and meta‐dehumanization research findings, which have relied almost exclusively on measures that do not meet the COSMIN standards for measurement quality. Ongoing efforts to validate existing measures and replicate research findings are recommended to improve the quality of evidence in this emerging field.

There were several general limitations of all other measures included in this review. First, high‐quality content validity studies were seldom considered in measure development, resulting in ‘very low’ quality evidence (based on reviewer ratings) for all but two (Golossenko et al., 2023; Sakalaki et al., 2017) of the measures. This is not to say the quality of content validity is low – reviewer ratings deemed many of the measures as having sufficient content validity properties – but instead, the quality of evidence for this is low; reviewer ratings are simply the perception of the authors as to whether a measure has sufficient content validity. For high‐quality content validity ratings, measures must involve the perceptions of the target population in determining item relevance, comprehension and comprehensibility, alongside professional opinions. Mokkink et al. (2024) state content validity is the most important psychometric property to be considered when selecting which outcome measure to choose. In the present review, most measures did not involve the target population and professionals in their development. The importance of service user involvement within mental health research is widely recognized (Sangill et al., 2019) and strongly advocated for within the field of applied psychology. Measures have generally been constructed by applying theory and items from (other‐based) dehumanization scales to the self, or by consulting researchers only. An assumption is made about how self‐ and meta‐dehumanization is experienced without input from people who identify as feeling dehumanized. This is a significant issue which could result in substantial measurement errors. Meaningful involvement of the target population and professionals within item selection must be implemented in future measure development (e.g. through consultations, focus groups, Delphi study, semi‐structured interviews, cognitive interviews, pilot testing) to improve content validity of measures.

Second, a significant number of measures have not undergone structural validity tests, meaning that it is unclear whether their factorial structure aligns with theoretical dimensions. This is especially important, as measures of meta‐dehumanization have demonstrated one (Demoulin et al., 2021), two (Bastian et al., 2013) and four (Pizzirani et al., 2019) factor solutions; thus, it is unclear what an expected factor structure of such a measure should be. Structural validity is important when considering whether a measure generalizes to a different population than that in which it was initially developed. Structural validity can be assessed through exploratory and confirmatory factor analyses on an adequate sample size (Mokkink, Prinsen, et al., 2018). Evidence of factor structure would also improve internal consistency ratings, as internal consistency, according to COSMIN guidance (Mokkink, Prinsen, et al., 2018) is dependent upon at least some evidence of factor structure. Future research should endeavour to establish the structural validity of any new and existing measures of self‐ and meta‐dehumanization.

Thirdly, all but two (Cheung, 2024; Sakalaki et al., 2017) self‐ and meta‐dehumanization measures assessed test–retest reliability. Test–retest reliability gives an indication of the stability of a measure over a short period of time and is especially important for measures being used in longitudinal research. Test–retest reliability can be measured via an intraclass correlation coefficient or a Pearson's correlation between two briefly spaced time points. Reliability testing of new and existing measures of self‐ and meta‐dehumanization is another direction for future research. Other measurement properties which remain under‐investigated include measurement invariance, responsiveness and measurement error.

Finally, measures of self‐ and meta‐dehumanization drew from a wide range of theoretical backgrounds. This review purposefully chose broad inclusion criteria, reviewing measures for different contexts and populations, and considered all forms of the felt sense of dehumanization. The most prominent theoretical underpinning was Haslam's (2006; latterly Bastian & Haslam, 2010) conception of dehumanization, comprising animalistic and mechanistic states; a denial of one's human nature and human uniqueness. Other theories drawn upon in measures include the Mind Perception account posited by Kouchaki et al. (2018), with the implication being that to self‐dehumanize is to deny oneself a mind. Infrahumanization theory implies that self‐infrahumanization is the denial of secondary emotional states to oneself (Leyens et al., 2000, 2001; McCleary‐Gaddy & James, 2022). Self‐objectification involves likening oneself to an object (Talmon & Ginzburg, 2016). While authors constructing these scales used different names (e.g. objectification, dehumanization), they have all been applied to measure the felt sense of dehumanization. Researchers should carefully consider which measures they are selecting for use and transparently report their theoretical underpinnings. A lack of clarity around this risks the jingle‐jangle fallacy: when measures of the same name measure different constructs, and when measures of a different name measure the same construct (Flake & Fried, 2020). This has been observed in other psychological phenomena, including mindfulness (Altgassen et al., 2024) and empathy (Hall & Schwartz, 2022). We echo the call by Hayden (2022) for consensus around theoretical models and underpinnings in measure development. Not doing so risks multiple measures attempting to capture dehumanization in slightly different ways, which could hinder efforts for reproducibility and validity of research findings.

### Limitations

COSMIN guidance has previously been deemed overly conservative (Smith et al., 2021). This was found to be the case in this review; for example, in requiring both target population and professional involvement for the measure to have anything other than very low‐quality evidence of content validity. As a result, measures which received input from external professionals during item development would receive the same quality rating as those in which items were generated by the authorship team only. Another limitation of COSMIN guidance is that many of the measures included in this review were developed in areas of psychology (e.g. social psychology) where adherence to these guidelines is less common. It might seem unreasonable to apply such a standards to these measures, but as self‐ and meta‐dehumanization are increasingly being explored in the context of clinical psychology (Jenkins et al., 2023), it is important that measures meet established standards of psychometric rigour. Further, it is a limitation of this review that measures written in languages other than English were not included. Dehumanization measures in Arabic (Abou Zeid et al., 2024), Chinese (Jiang et al., 2021), Italian (Cervone et al., 2025) and Spanish (Ariño‐Mateo et al., 2022) do exist, but it was beyond the scope of this review to assess their measurement properties.

### Conclusion

This is the first article to systematically review available self‐report questionnaires measuring self‐dehumanization and meta‐dehumanization. There were a variety of theoretical frameworks underpinning these measures. Quality assessment of the 14 self‐dehumanization measures and 15 meta‐dehumanization measures using the COSMIN guidance revealed generally very low‐quality evidence for content validity – notably through a lack of target population and professional involvement in development, and a greater need to evaluate other measurement properties including structural validity and reliability. Only one measure, the Experience of Dehumanization Measure (Golossenko et al., 2023) is recommended for use at present. As self‐ and meta‐dehumanization are being increasingly recognized in clinical practice, it is essential that gold standard outcome measures relevant for specific clinical populations are created following the best practice recommendations (Boateng et al., 2018; Mokkink et al., 2024), alongside ongoing efforts to validate existing measures.

## AUTHOR CONTRIBUTIONS

**Tom A. Jenkins:** Writing – original draft; conceptualization; investigation; methodology; writing – review and editing; formal analysis; project administration. **Hannah Pendlebury:** Project administration; formal analysis; methodology; writing – review and editing; writing – original draft; investigation; conceptualization. **Spencer L. Smith:** Writing – review and editing; formal analysis; investigation.

## Supporting information


Data S1.

## Data Availability

Data sharing not applicable to this article as no datasets were generated or analysed during the current study.
