# Supplementary material for: Measuring the felt sense of dehumanization: A COSMIN systematic review of the psychometric properties of self‐ and meta‐dehumanization measures
Source: Br J Psychol. 2025 Aug 8;117(1):155–76. doi: 10.1111/bjop.70017 (PMC12783879; doi:10.1111/bjop.70017)
Supplement: Supplementary file 1 — Data S1. [file BJOP-117-155-s001.docx]

**Appendix A:** Literature Review - Search strategy for each database

**Web of Science**

(((((TI=(dehuman*)) OR AB=(dehuman*)) OR TI=(infrahuman*)) OR AB=(infrahuman*)) OR TI=("internalizing objectification")) OR AB=("internalizing objectification")

**PubMed**

**((dehuman*[Title/Abstract]) OR (infrahuman*[Title/Abstract])) OR ("internalizing objectification"[Title/Abstract])**

**PsycINFO**

Journal Title: dehuman* *OR* Abstract: dehuman* *OR* Journal Title: infrahuman* *OR* Abstract: infrahuman* *OR* Journal Title: "internalizing objectification" *AND* Abstract: "internalizing objectification" *AND* Peer-Reviewed Journals only

**Scopus**

( TITLE-ABS-KEY ( dehuman* ) OR TITLE-ABS-KEY ( infrahuman* ) OR TITLE-ABS-KEY ( "internalizing objectification" ) ) AND ( LIMIT-TO ( DOCTYPE , "ar" ) )

**Appendix B:** Literature Review – PRISMA reporting guidelines

| **Section and Topic** | **Item #** | **Checklist item** | **Location where item is reported** |
| --- | --- | --- | --- |
| **TITLE** | | |  |
| Title | 1 | Identify the report as a systematic review. | Title page |
| **ABSTRACT** | | |  |
| Abstract | 2 | See the PRISMA 2020 for Abstracts checklist. | Abstract |
| **INTRODUCTION** | | |  |
| Rationale | 3 | Describe the rationale for the review in the context of existing knowledge. | Introduction |
| Objectives | 4 | Provide an explicit statement of the objective(s) or question(s) the review addresses. | Review aim |
| **METHODS** | | |  |
| Eligibility criteria | 5 | Specify the inclusion and exclusion criteria for the review and how studies were grouped for the syntheses. | Eligibility criteria |
| Information sources | 6 | Specify all databases, registers, websites, organisations, reference lists and other sources searched or consulted to identify studies. Specify the date when each source was last searched or consulted. | Search strategy |
| Search strategy | 7 | Present the full search strategies for all databases, registers and websites, including any filters and limits used. | Appendix A |
| Selection process | 8 | Specify the methods used to decide whether a study met the inclusion criteria of the review, including how many reviewers screened each record and each report retrieved, whether they worked independently, and if applicable, details of automation tools used in the process. | Procedure |
| Data collection process | 9 | Specify the methods used to collect data from reports, including how many reviewers collected data from each report, whether they worked independently, any processes for obtaining or confirming data from study investigators, and if applicable, details of automation tools used in the process. | Data Extraction and Quality Assessment |
| Data items | 10a | List and define all outcomes for which data were sought. Specify whether all results that were compatible with each outcome domain in each study were sought (e.g. for all measures, time points, analyses), and if not, the methods used to decide which results to collect. | Data Extraction and Quality Assessment |
|  | 10b | List and define all other variables for which data were sought (e.g. participant and intervention characteristics, funding sources). Describe any assumptions made about any missing or unclear information. |  |
| Study risk of bias assessment | 11 | Specify the methods used to assess risk of bias in the included studies, including details of the tool(s) used, how many reviewers assessed each study and whether they worked independently, and if applicable, details of automation tools used in the process. | Quality Assessment |
| Effect measures | 12 | Specify for each outcome the effect measure(s) (e.g. risk ratio, mean difference) used in the synthesis or presentation of results. | Quality Assessment |
| Synthesis methods | 13a | Describe the processes used to decide which studies were eligible for each synthesis (e.g. tabulating the study intervention characteristics and comparing against the planned groups for each synthesis (item #5)). | Not applicable: no evidence synthesis conducted |
|  | 13b | Describe any methods required to prepare the data for presentation or synthesis, such as handling of missing summary statistics, or data conversions. |  |
|  | 13c | Describe any methods used to tabulate or visually display results of individual studies and syntheses. |  |
|  | 13d | Describe any methods used to synthesize results and provide a rationale for the choice(s). If meta-analysis was performed, describe the model(s), method(s) to identify the presence and extent of statistical heterogeneity, and software package(s) used. |  |
|  | 13e | Describe any methods used to explore possible causes of heterogeneity among study results (e.g. subgroup analysis, meta-regression). |  |
|  | 13f | Describe any sensitivity analyses conducted to assess robustness of the synthesized results. |  |
| Reporting bias assessment | 14 | Describe any methods used to assess risk of bias due to missing results in a synthesis (arising from reporting biases). |  |
| Certainty assessment | 15 | Describe any methods used to assess certainty (or confidence) in the body of evidence for an outcome. | Not applicable |
| **RESULTS** | | |  |
| Study selection | 16a | Describe the results of the search and selection process, from the number of records identified in the search to the number of studies included in the review, ideally using a flow diagram. | Results; flow diagram (Figure 1) |
|  | 16b | Cite studies that might appear to meet the inclusion criteria, but which were excluded, and explain why they were excluded. |  |
| Study characteristics | 17 | Cite each included study and present its characteristics. | Results; Table 1 |
| Risk of bias in studies | 18 | Present assessments of risk of bias for each included study. | Results; Table 2; Appendix C |
| Results of individual studies | 19 | For all outcomes, present, for each study: (a) summary statistics for each group (where appropriate) and (b) an effect estimate and its precision (e.g. confidence/credible interval), ideally using structured tables or plots. | Results; Table 2 |
| Results of syntheses | 20a | For each synthesis, briefly summarise the characteristics and risk of bias among contributing studies. |  |
|  | 20b | Present results of all statistical syntheses conducted. If meta-analysis was done, present for each the summary estimate and its precision (e.g. confidence/credible interval) and measures of statistical heterogeneity. If comparing groups, describe the direction of the effect. | Not applicable: no evidence synthesis conducted |
|  | 20c | Present results of all investigations of possible causes of heterogeneity among study results. |  |
|  | 20d | Present results of all sensitivity analyses conducted to assess the robustness of the synthesized results. |  |
| Reporting biases | 21 | Present assessments of risk of bias due to missing results (arising from reporting biases) for each synthesis assessed. |  |
| Certainty of evidence | 22 | Present assessments of certainty (or confidence) in the body of evidence for each outcome assessed. | Not applicable |
| **DISCUSSION** | | |  |
| Discussion | 23a | Provide a general interpretation of the results in the context of other evidence. | Discussion |
|  | 23b | Discuss any limitations of the evidence included in the review. | Discussion |
|  | 23c | Discuss any limitations of the review processes used. | Discussion: Limitations |
|  | 23d | Discuss implications of the results for practice, policy, and future research. | Discussion |
| **OTHER INFORMATION** | | |  |
| Registration and protocol | 24a | Provide registration information for the review, including register name and registration number, or state that the review was not registered. | Method |
|  | 24b | Indicate where the review protocol can be accessed, or state that a protocol was not prepared. | Method |
|  | 24c | Describe and explain any amendments to information provided at registration or in the protocol. | Method |
| Support | 25 | Describe sources of financial or non-financial support for the review, and the role of the funders or sponsors in the review. | Not applicable |
| Competing interests | 26 | Declare any competing interests of review authors. | Not applicable |

**Appendix C**

**Study characteristics**

| **Study Author** | **Measure** | **Sample size** | **Gender Percentage** | **Mean age (range)** | **Location** | **Psychometrics reported** |
| --- | --- | --- | --- | --- | --- | --- |
| Bastian and Haslam (2010) | *Self-Dehumanisation Measure (2010)* | 72 | 36% Female (46) | 19.21 (17-52) | Australia | Internal consistency, construct validity |
| Bastian et al. (2013) | *Measure of Self-Humanity (2013)* | 84 | 64.3% Female (54) | 23.04 | Australia | Structural validity, internal consistency, construct validity |
| Baldissarri et al. (2017) | *Self-perception of Being Instrument-like vs. Human-like (2017)* | 102 | 69.6% Female (71) | 22.76 (18-63) | Italy | Internal consistency, construct validity |
| Baldissarri et al. (2014) | *Self-Mental State Attribution Task in the Workplace (2014)* | 120 | 37% Female (44) | 32.79 (19-56) | Italy | Internal consistency, construct validity |
|  | *Perception of Being Objectified by Supervisors (PBOS; 2014)* | 120 | 37% Female (44) | 32.79 (19-56) | Italy | Internal consistency, construct validity |
| Fontesse, Demoulin, et al. (2021) | *Self-dehumanization Scale in Patients with Severe Alcohol Use Disorder (2021)* | 110 | 27% Female (30) | 48.3 | Belgium | Internal consistency, construct validity |
| Kouchaki et al. (2018) | *Self-Dehumanisation Measure Adapted from Mind Attribution Scale (2018)* | 221 | 37.1% Female (82) | 29.2 | USA | Internal consistency, construct validity |
| Sakalaki et al. (2017) | *Mechanistic Self-Dehumanisation Scale (2017)* | 158 | 60% Female (94) | 18-60 | Greece | Internal consistency, test re-test reliability, construct validity |
| Roupa et al. (2024) | *Mechanistic Self-Dehumanisation Scale (2017)* | 400 | 74.3% female (297) | 43.8 | Greece | Structural validity, internal consistency, content validity |
| Talmon and Ginzburg (2016) | *Self-Objectification Scale (2016)* | 373  300 | 54% Female (200)  100% Female (300) | 25.12  25.01 | Israel | Internal consistency, structural validity Construct validity, criterion validity, structural validity |
| McCleary-Gaddy and James (2022) | *Author-constructed Self-Infrahumanization Scale in African American Women (2022)* | 449 | 100% Female (449) | 18-87 | USA | Internal consistency, construct validity |
| Sakalaki et al. (2016) | *Low Human Nature Traits Scale (2016)* | 109 | 59% Female (64) | 18-60 | Greece | Internal consistency, construct validity, criterion validity |
| Caesens et al. (2017) | *Organisational Dehumanisation Measure (2017)* | 1209 | 48.97% Female (592) | 38.93 | Belgium | Internal consistency, structural validity, construct validity |
| Cheung (2024) | *Organisational animalistic dehumanization scale* | 574 | 44.8% Female (257) | 18-65+ | USA | Internal consistency, construct validity, structural validity, test re-test reliability |
| Lagios et al. (2024) | *Organisational Dehumanisation Measure Short* | 1209 | 48.97% Female (592) | 38.93 | Belgium | Internal consistency, structural validity, criterion validity, construct validity |
| Kteily et al. (2016) | *Meta-dehumanisation Measure (2016)* | 2498 | 51% Female (1265) | 35.45 | USA, Hungary, Israel | Internal consistency, construct validity |
| Fontesse et al. (2020) | *Metadehumanization scale in Patients with Severe Alcohol Use Disorder (2020)* | 120 | 28% Female (34) | 48.3 | Belgium | Internal consistency, construct validity |
| Bastian and Haslam (2011) | *Meta-dehumanization Measure (2011)* | 69 | 74% Female (51) | 19.18 (17-37) | Australia | Internal consistency, structural validity, construct validity |
| Demoulin et al. (2021) | *Metadehumanization in Women Measure (2021)* | 325 | 100% Female (325) | 36.5 | UK & USA | Internal consistency, construct validity, structural validity |
|  | *Metadehumanization in people with severe alcohol use disorder measure (2021)* | 102 | 30% Female (30) | 33.81 | UK & USA | Internal consistency, construct validity, structural validity |
| Punchihewa & Broadbent (2024) | *Patient gown dehumanisation questionnaire* | 74 | 50% Female (37) | N/A | New Zealand | Internal consistency, construct validity |
| Auzoult and Personnaz (2016) | *Perception of Being Objectified in the Workplace Scale (2016)* | 363 | 47% Female (169) | 44.4 | France | Internal consistency, construct validity, structural validity |
| Crone and Brunel (2021) | *Perception of Objectification in the Workplace Short Scale* | 780  74 | 51% Female (398)  82.4% Female (61) | 38  37 | France | Internal consistency, structural validity  Construct validity |
| Cervone et al. (2025) | *Meta-dehumanisation from sexism scale* | 423 | 100% Female (423) | 31.91 (19-72) | UK and Italy | Internal consistency, construct validity |
|  | *Self-infrahumanisation from sexism scale* | 423 | 100% Female (423) | 31.91 (19-72) | UK and Italy | Internal consistency, construct validity |
|  | *Self-objectification from sexism scale* | 423 | 100% Female (423) | 31.91 (19-72) | UK and Italy | Internal consistency, construct validity |
| Golossenko et al. (2023) | *Experience of Dehumanisation Measure (2023)* | 41  710  248  332  302  283  207 | 61% Female (25)  66.9% Female (475)  66.53% Female (165)  58.43% Female (194)  71.85% Female (217)  56.89% Female (161)  58.45% Female (121 | 34.35  39.02  34.41  29.56  32.65  39.09  37.36 | UK & Spain  UK  UK  UK  UK  UK  UK | Content validity  Structural validity, internal consistency  Internal consistency, structural validity  Internal consistency, construct validity  Internal consistency, construct validity  Internal consistency, construct validity  Internal consistency, construct validity |
| Robison et al. (2025) | *Self Dehumanization Scale (2025)* | 482  388  304 | 72.8% Female (351)  57.2% Female (222)  78% Female (237) | 19.24 (18-25)  34.81 (18-72)  19.89 (18-39) | USA  USA  USA | Internal consistency, construct validity, structural validity  Internal consistency, construct validity, structural validity  Internal consistency, construct validity, structural validity |
| Pizzirani et al. (2019) | [*Dehumanization within Romantic Relationships*](https://www.frontiersin.org/journals/psychology/articles/10.3389/fpsyg.2019.02754/full) *Scale (2019)* | 1251  847  328 | 60.1% Female (752)  61.4% Female (520)  72% Female (236) | 25.35 (18-63)  25.55 (18-59)  23.40 (18-60) | USA, Canada, Australia, UK, New Zealand | Internal consistency, structural validity  Structural validity, construct validity  Structural validity, construct validity |
| Robison et al. (2024) | *Measure of Perceived Dehumanization from Officers (2024)* | 411  3531 | 28% Female (115)  31% Female (1095) | 35  38.8 | USA | Internal consistency, structural validity  Internal consistency, structural validity  construct validity |
